# Effects of functional dietary fiber supplementation combined with home-based exercise on gut microbiota diversity and low-grade inflammation in urban sedentary adults

**DOI:** 10.3389/fnut.2026.1769785

**Published:** 2026-03-04

**Authors:** Wei Wang, Ye Tao, Mengke Zhu

**Affiliations:** Department of Physical Education, Sejong University, Seoul, Republic of Korea

**Keywords:** functional dietary fiber, gut microbiota diversity, home-based exercise, low-grade inflammation, sedentary behavior

## Abstract

**Introduction:**

Sedentary behavior is associated with gut microbiota dysbiosis and low-grade systemic inflammation, both of which contribute to increased cardiometabolic risk. However, the combined effects of functional dietary fiber supplementation and home-based exercise on these outcomes remain unclear. This study aimed to investigate whether a combined intervention could improve gut microbiota diversity and reduce systemic inflammation in urban sedentary adults.

**Methods:**

In this 24-week parallel-group randomized controlled trial, 140 sedentary adults were randomly assigned to an intervention group (functional dietary fiber supplementation providing 15–20 g/day of resistant starch, inulin, and beta-glucan combined with home-based moderate-intensity exercise, five sessions per week) or a control group maintaining their usual lifestyle. Gut microbiota diversity was assessed using 16S rRNA gene sequencing, and inflammatory markers (hs-CRP, IL-6, TNF-α, IL-10) were measured using immunoassays.

**Results:**

The intervention significantly increased gut microbiota alpha diversity, with Shannon index rising from 3.82 ± 0.48 to 4.31 ± 0.49 (*p* < 0.001), while minimal changes were observed in controls. Significant reductions were observed in hs-CRP (−42.1%), IL-6 (−35.4%), and TNF-α (−28.6%), alongside an increase in IL-10 (+31.8%) (all *p* < 0.001). Butyrate levels increased by 50%, and changes in Shannon diversity were negatively correlated with reductions in hs-CRP (*r* = −0.52, *p* < 0.001).

**Conclusion:**

Combined functional dietary fiber supplementation and home-based exercise significantly improved gut microbiota diversity and reduced low-grade inflammation in sedentary adults. These findings support integrated lifestyle interventions as effective strategies for reducing cardiometabolic risk.

## 1 Introduction

The pace of the current era characterized by rapid urbanization and technology adoption has drastically altered human mobility habits, and sedentary behavior has appeared in the spotlight as the hallmark of modern living habits (1). Increases in sitting time, generally considered as sitting for eight or more hours per day, have increased with alarming rates in office goers and working professionals around the globe (2). The World Health Organization has identified sedentary behavior as an independent risk factor for undesirable outcomes, which led to the creation of certain guidelines related to physical activity and sedentary behavior (3). It has been found in epidemiological studies that there is convincing evidence of increased risk of events like cardiovascular disease, type 2 diabetes mellitus, and all-cause deaths due to sedentary behavior, despite achieving satisfactory physical activity level requirements (4). These relationships remained significant after adjustment in assumed confounding variables, underlining the need to tackle sedentary population effectively (5).

The core condition in the pathophysiology of sedentary behavior and chronic disease is the process of low-grade systemic inflammation, where there is moderate but constant elevation in the level of systemic inflammatory factors (6). This chronic inflammatory state is intricately linked to the development of metabolic syndrome, a cluster of conditions including central obesity, insulin resistance, dyslipidemia, and hypertension (7). The gut microbiota plays a pivotal role in this inflammation-metabolic syndrome axis through multiple mechanisms. Dysbiosis, characterized by reduced microbial diversity and altered bacterial composition, can lead to increased intestinal permeability (commonly termed “leaky gut”), facilitating the translocation of bacterial lipopolysaccharides (LPS) into systemic circulation (8). This endotoxemia triggers Toll-like receptor 4 (TLR4) activation on immune cells, initiating pro-inflammatory cascades that contribute to insulin resistance, hepatic steatosis, and adipose tissue inflammation (9). Furthermore, alterations in gut microbial metabolites, particularly reduced short-chain fatty acid (SCFA) production, impair the regulatory functions of intestinal epithelial cells and immune homeostasis, creating a self-perpetuating cycle of inflammation and metabolic dysfunction (10). In contradistinction to acute inflammation, where there is protective mechanism involvement, low-grade inflammation impacts negatively in the long run through endothelial dysfunction, increased insulin resistance, and consequent injury to tissues. The high-sensitivity C-reactive protein, interleukin-6, and tumor necrosis factor alpha have all proved to be valid biomarkers of inflammation, where increased protein levels predict future cardiometabolic events (7). Studies have shown that increased sitting times increase inflammation through increased adiposity, decreased muscle metabolic rates, and glucose intolerance (11). It has further emerged that lifestyle modifications through food changes and exercise can effectively reduce these biomarkers of inflammation, suggesting that inflammation can potentially be treated as a target in disease prevention (12).

The gastrointestinal microbiome has received much attention as an important mediator of host metabolic and immune function (8). A healthy and diverse gastrointestinal microbiome has shown benefits in nutrient metabolism, protection against pathogens, and immune system development, whereas an imbalance has been shown to contribute to many chronic diseases (9). The gastrointestinal microbiome-inflammation axis has emerged as an important pathway through which lifestyle factors affect host health. An imbalance in host microbiomes can lead to impaired barrier function in the gastrointestinal tract, thereby facilitating bacterial lipopolysaccharide translocation from the gastrointestinal tract into systemic circulation, resulting in inflammation. In other words, the gastrointestinal microbiome has emerged as both an important biomarker and target in host health.

Dietary fiber, especially the fermentable type, has intense influences on microbial composition and function in the gastrointestinal tract (13). The specific fiber blend used in this study—resistant starch type 2 (RS2), inulin, and beta-glucan—was strategically selected based on their distinct and complementary prebiotic mechanisms. RS2 resists digestion in the small intestine and reaches the colon intact, where it serves as a selective substrate for Bifidobacterium and butyrate-producing bacteria such as *Faecalibacterium prausnitzii* and Roseburia species (14). Inulin, a fructan-type fiber, preferentially promotes the growth of Bifidobacterium species and enhances the production of acetate and propionate through fermentation (15). Beta-glucan from oats contributes to viscosity modulation in the gut environment and has been shown to stimulate the growth of Lactobacillus species while also modulating immune responses through interaction with dectin-1 receptors on intestinal immune cells (16). This multi-component approach was designed to target different segments of the colonic microbiota and maximize prebiotic efficacy across diverse bacterial populations. Being the main fermentation substrate in the colon, dietary fiber contributes to the increase in beneficial microbes and the synthesis of SCFAs such as acetate, propionate, and butyrate (17). These compounds function as fuel for colon cells, improve barrier function, and affect immune regulation through binding to G-protein coupled receptors. Systematic reviews have demonstrated the increase in microbial diversity and SCFAs in healthy adults with increased dietary fiber consumption (18). Newer evidence has further explored the role of bacterial metabolites in suppressing inflammation and identified butyrate as having intense anti-inflammatory activity through its ability to inhibit histone deacetylases and reduce NF-kappaB (10).

Physical exercise is another strong modifier of the gut microbiome ecosystem (19). Comparative analysis demonstrates that there are significant differences in microbiome composition between exercise-trained and sedentary subjects, where exercise-trained subjects have increased diversity in addition to increased concentrations of beneficial microbes. Exercise affects the microenvironment in several ways, such as increased motility in the intestines, altered bile acid secretion, and increased blood flow in the gastrointestinal tract (20). Ecological niches are thereby established that are favorable to beneficial microbes in addition to reducing inflammation in the system due to myokine secretion. Ecological niches are thereby established that are favorable to beneficial microbes in addition to reducing inflammation in the system due to myokine secretion (21). Notably, exercise intensity plays a critical role in determining health outcomes. While moderate-intensity exercise has been consistently associated with beneficial effects on gut microbiota diversity, immune function, and inflammatory markers, excessive or prolonged high-intensity exercise can paradoxically increase intestinal permeability, induce transient endotoxemia, and elevate pro-inflammatory cytokines (22). This dose–response relationship underscores the importance of exercise prescription optimization. The home-based exercise protocol employed in this study was specifically designed to maintain moderate intensity, defined as 12–14 on the Borg Rating of Perceived Exertion (RPE) scale, corresponding to approximately 50–70% of maximum heart rate. This intensity range has been demonstrated in previous studies to maximize anti-inflammatory benefits while minimizing the risk of exercise-induced gastrointestinal stress or immune suppression (23, 24).

Although there has been substantial evidence supporting individual benefits from dietary fiber supplements and physical exercise, there exist large gaps in understanding the combined interactions (25). Most of these studies have focused on individual aspects, thereby hindering any combined interactions that might exist. Cronin et al. reported modest microbiota improvements with exercise plus protein supplementation (26), and Allen et al. showed exercise alone alters microbiota composition (27), but comprehensive combined prebiotic-exercise trials in sedentary adults remain limited. In addition, there have been few studies focused on sedentary cities despite the fact that such communities portray high-risk rates towards inflammation-driven chronic diseases. Practicality in translating exercise programs from laboratories to actual settings can also pose challenges based on facility requirements that might hinder exercise scalability. Home exercise programs can serve as suitable alternatives; however, there has been inadequate examination at the combined aspects of these programs related to dietary considerations.

The current research works to overcome these issues by conducting a randomized controlled trial examining the combined treatment effects of functional dietary fiber supplement intake and a home-based exercise program in sedentary adults from an urban setting. The treatment utilizes a new fiber supplement composition comprising resistant starch, inulin, and beta-glucan, working in tandem to fully leverage prebiotic activity against diversified bacterial loads. The exercise program follows a progressive protocol that can be accomplished in a home setting using limited exercise equipment. The current trial seeks to uncover the functional associations between microbial and inflammation-related endpoints by concurrently examining both variables. The primary objectives were to evaluate whether the combined intervention would: (1) increase gut microbiota alpha diversity (Shannon index), and (2) reduce systemic inflammation (hs-CRP). Secondary objectives included other inflammatory markers, body composition, and quality of life. Exploratory objectives examined SCFA changes and mediation pathways. These results can help offer practical recommendations regarding lifestyle modifications aimed at the increasingly represented sedentary subgroup in the population.

## 2 Methods

### 2.1 Study design and ethics

This study was a 24-week, parallel-group randomized controlled trial with an additional midpoint assessment at week 12. Participants were prospectively recruited from April 2023 to June 2023. Random allocation was performed in a 1:1 ratio using a computer-generated stratified block randomization sequence. Due to the behavioral nature of the intervention, participant blinding was not feasible; however, outcome assessors remained blinded to group allocation.

The study protocol was prospectively registered at ClinicalTrials.gov (Identifier: NCT05987341) prior to participant enrollment. Ethical approval for the study was obtained from the Institutional Review Board of Sejong University, Republic of Korea (IRB Approval No. SJU-IRB-2023-117). All participants received verbal and written information about the study procedures, risks, and benefits, and written informed consent was obtained from each participant prior to baseline assessments. All procedures were conducted in accordance with the Declaration of Helsinki and are reported following CONSORT guidelines.

### 2.2 Participants

Participants were recruited from April 2023 to June 2023 through company partnerships, community advertisements, social media announcements, and referrals from outpatient clinics. The target population consisted of sedentary adults whose occupational and lifestyle patterns involved prolonged sitting. The target population was sedentary adults whose lifestyle and employment activity placed them at increased risk of sitting for longer periods of time with little exercise.

Eligibility criteria included being between 25 and 55 years of age, having at least 8 h per day of sitting time, less than 150 min of moderate-intensity activity per week based on data from accelerometers or the International Physical Activity Questionnaire, and having a BMI of 18.5 to 30 kg/m^2^. Exclusion criteria included taking any course of antibiotics in the last 3 months, having diagnoses of Inflammatory Bowel Disease, Irritable Bowel Syndrome, Autoimmune disorders, Diabetes, or Severe Cardiovascular Disease, being pregnant or breastfeeding, taking Prebiotics or Probiotics regularly as supplements, or having any physical limitations that make it difficult to participate in exercise at home. All subjects gave written, informed consent pre-baseline.

### 2.3 Sample size estimation

The calculation of sample size was performed using the primary outcome variable, which was the Shannon diversity index of the gut microbiota. Assuming an increase of 0.3 units in the Shannon diversity index with a standard deviation of 0.5 based on the previously reported data from related literature, the estimated effect size was found to be Cohen’s d = 0.6. To attain the specified power of 80% with a two-sided test and fixed—Alpha = 0.05, the sample size was calculated using the G*Power calculation software version 3.1.9.7. To counteract the estimated dropout of 20% in the 24-week treatment period, the total number of participants was estimated to be around 70 in each group, leading to a final sample size of 140.

### 2.4 Intervention protocol

Patients in the treatment group concurrently took functional dietary fiber supplements and exercised at home in accordance with the program strictly for 24 weeks. The fiber supplement was comprised of resistant starch type 2 (RS2) at 40%, inulin at 30%, and beta-glucan from oats at 30%, in a 15-20 g total daily dose. This 40%/30%/30% ratio was designed to target complementary bacterial populations while maintaining tolerability, as higher single-fiber doses can increase gastrointestinal discomfort (18). For optimal gastrointestinal tolerance, the dose was begun at 10 g per day in the first 2 weeks with progressive increase of the total dose thereafter. The supplement was taken in two equally divided portions with breakfast and dinner, dissolved in water, milk, or yogurt.

The exercise part included five exercise sessions per week, ranging from 30 to 45 min at moderate intensity (RPE 12–14, corresponding to 50–70% of age-predicted maximum heart rate). Exercise adherence was monitored through dual verification: (1) wrist-worn heart rate monitors (Xiaomi Mi Band 7) recording exercise duration and heart rate data synchronized to a study mobile application; and (2) daily exercise logs documenting session duration and self-reported RPE. The correlation between objective and self-reported data was *r* = 0.78 (*p* < 0.001). Every exercise session included the standard exercise protocol: dynamic warm-up exercise, aerobic exercise in the form of marching and jumping, weight resistance exercises such as squats and push-ups, and closing stretches. Control participants maintained usual lifestyle and received a general health education booklet covering sleep and stress management, without specific recommendations on dietary fiber or structured exercise. Behavioral stability was confirmed: fiber intake (12.8 to 13.2 g/day, *p* = 0.42) and physical activity (58.6 to 64.2 min/week, *p* = 0.31) remained unchanged. Detailed exercise protocol specifications are provided in Supplementary Material S1.

### 2.5 Outcome measures and assessment

The main endpoints included diversity of the gut microbiota and systemic inflammation. Microbiota analysis was performed using alpha diversity metrics including Shannon index, Chao1, and Simpson index, in addition to beta diversity indices estimated by means of Bray–Curtis dissimilarity and weighted UniFrac distances. The taxonomical analysis was further performed at the level of phylum, family, and genus using 16S rRNA gene sequencing, targeting the V3-V4 region using primers 341F/806R, sequenced on Illumina NovaSeq 6,000 (2 × 250 bp). Bioinformatic processing used QIIME2 with DADA2 and SILVA 138.1 database for taxonomy. Inflammation was further determined by evaluating the level of high-sensitivity C-reactive protein, interleukin-6, tumor necrosis factor alpha, and the anti-inflammatory cytokine interleukin-10 in the serum hs-CRP was measured using particle-enhanced immunoturbidimetric assay (Roche Cobas c702). IL-6, TNF-*α*, and IL-10 were measured using electrochemiluminescence immunoassay (Roche Cobas e801). Zonulin was measured using competitive ELISA (Immundiagnostik AG).

Secondary endpoints included the following parameters of body composition: fat percent and lean mass, both measured by bioelectrical impedance analysis; other parameters such as body weight, body mass index, waist circumference, and waist-hip ratio, all measured by direct anthropometry; and sedentary activity accumulated per day as recorded by accelerometry and the IPAQ. Also included as endpoints was the measurement of subjective fatigue using the MFI, and the quality of life using the SF-36. All endpoints were tested at baseline, week 12, and week 24.

Exploratory mechanistic outcomes included fecal short-chain fatty acid concentrations (acetate, propionate, and butyrate) quantified by GC–MS (Agilent 7890B-5977B) with a DB-FFAP column. Samples were acidified, extracted with diethyl ether, derivatized with BSTFA, and quantified using authentic SCFA standards. Intestinal barrier function was assessed through serum zonulin concentrations measured by enzyme-linked immunosorbent assay (ELISA) and lipopolysaccharide-binding protein (LBP) determined by immunoturbidimetric assay.

### 2.6 Data collection and quality control

Fecal samples were collected using a standard procedure and immediately frozen at −80 degrees Celsius. Venous blood was collected after an overnight fast of at least 8 hours. The dietary intake was collected using food records keeping for 3 days, including one weekend day. Adherence to the treatment was determined through app-based communication related to exercise activity assessment and return of empty supplement packets every week. Systematic collection of adverse events was reported all through the duration of the trial, using standard definitions related to severity. Study staff was trained thoroughly in advance of data collection to ensure that there was standardization across all data points, and regular data integrity checks were performed. Blood samples were rescheduled if participants reported acute infection, significant dietary deviations, or anti-inflammatory medication use within 72 h prior to collection. Fecal samples were excluded if participants experienced acute gastrointestinal illness within 2 weeks.

### 2.7 Statistical analysis

All data analysis was done based on the intention-to-treat principle, with per-protocol analysis as a sensitivity analysis. For continuous data, means and standard deviations or medians and interquartile ranges, depending on distribution, were reported, whereas categorical data was presented through frequencies and percentages. Comparison of data across the groups at different points in time was done using linear model analysis to determine the interaction between time and the groups. These analysis models considered the issue of missing data. Linear mixed-effects models included fixed effects for group, time, group×time interaction, and covariates (age, sex, baseline BMI, fiber intake), with random intercepts for subjects (lme4 package). Missing data were handled using multiple imputation (MICE, 50 datasets).

For microbiota data, permutational multivariate analysis of variance (PERMANOVA) was conducted with 9,999 permutations using the vegan package to compare beta diversity differences between groups, and linear discriminant analysis effect size was used to uncover differentially abundant bacterial members. The relationships between changes in the composition of gut microbiota and inflammation indexes were tested using Spearman correlation coefficients. The potential mediating roles of short-chain fatty acids and bacterial members in the association between the treatment and inflammation endpoints were further tested using the mediation package with quasi-Bayesian Monte Carlo simulation (5,000 iterations) and bias-corrected bootstrap resampling (5,000 samples) to estimate average causal mediation effects and proportion mediated. All models were mutually adjusted to covary age, sex, baseline body mass index, and habitual dietary fiber intake. All comparisons were corrected using the Benjamini and Hochberg false discovery rate adjustment procedure. Statistical significance was judged using a two-sided test with a *p* value <0.05. Statistical analysis was performed using R software, version 4.x, in conjunction with the packages “vegan” and “phyloseq” in addition to “lme4” in conjunction with SPSS 26.0.

## 3 Results

### 3.1 Participant enrollment and baseline characteristics

**Figure 1:**
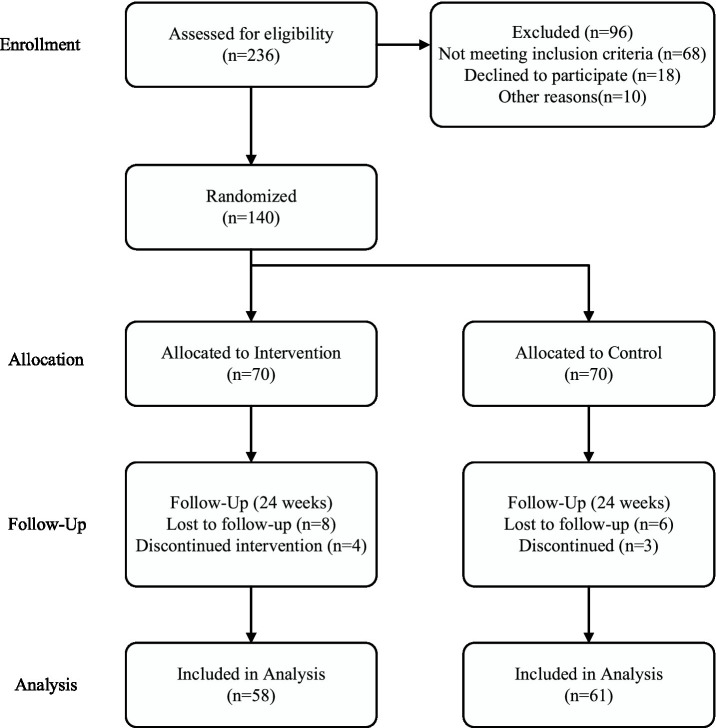
CONSORT flow diagram of participant enrollment, allocation, follow-up, and analysis.

In total, 236 persons expressed interest in taking part in this trial and proceeded with preliminary screening. After screening, 68 candidates were eliminated because they failed to meet the requirements. This was followed by other candidates who refused to participate in the trial after reviewing the requirements (18 candidates) and those who failed due to other reasons such as irregular lab results and scheduling conflicts (10 candidates). In the end, 140 candidates who seemed appropriate took part in the trial and was equally sampled in both the intervention and the control group (70 in each) as shown in the CONSORT flow diagram in Figure 1.

Over the course of the 24-week trial, 12 members of the treatment group and 9 members of the control group withdrew from the trial, giving rates of 82.9 and 87.1% retention, respectively. Dropouts in both treatment and control can be attributed to moving away, personal issues, adverse gastrointestinal reactions in the treatment group, and loss to follow-up. It can be asserted that the level of compliance in the treatment was good all through the duration of the trial. In terms of members who took part in the trial, the mean level of compliance with dietary fiber supplements was 87.4% ± 8.6%, as estimated from data regarding daily intake as reported in daily journals submitted or evidenced by return of empty packs. Regarding exercise, using application-based reminders, members’ mean level of compliance with exercise was 82.8% ± 10.2% of target sessions. In all, 68.9% of members in the treatment (*n* = 40) experienced high compliance (> = 80% compliance with both treatment components), whereas 31.1% (*n* = 18) experienced moderate compliance (60–79% compliance). Final analyses included members in the treatment who took all post-baseline measurements (58 members) and in the control (61 members).

Baseline demographic, anthropometric, and clinical parameters of both groups are shown in Table 1. The population was, on average, 38.6 years old, with females contributing 54.3% of the population. They reported sitting for approximately 9.2 h per day, and physical activity was low. Their average BMI was 24.8 kg/m^2^ (range: 18.6–29.8 kg/m^2^), and WC was 86.4 cm. The BMI distribution showed that 42.1% (*n* = 59) had BMI < 24 kg/m^2^ and 57.9% (*n* = 81) had BMI ≥ 24 kg/m^2^, with comparable distribution between groups (*p* = 0.743). Baseline inflammatory parameters such as high-sensitivity CRP, IL-6, and TNF-*α*, and alpha diversity of the gut microbiota, did not differ significantly between the groups, as shown in Table 1. These parameters helped ensure that any future differences found in the end points could be considered due to the treatment and not due to any differences at baseline.

**Table 1:** Baseline characteristics of study participants.

Variable	Intervention group (*n* = 70)	Control group (*n* = 70)	*p*-value
Demographics			
Age (years)	38.4 ± 8.2	38.8 ± 7.9	0.763
Sex, female (%)	37 (52.9)	39 (55.7)	0.731
Anthropometrics			
Body mass index (kg/m^2^)	24.7 ± 2.8	24.9 ± 2.6	0.654
Waist circumference (cm)	86.2 ± 9.4	86.6 ± 9.1	0.792
Body fat percentage (%)	28.4 ± 5.6	28.8 ± 5.3	0.661
Behavioral measures			
Daily sitting time (hours)	9.1 ± 1.4	9.3 ± 1.5	0.413
Dietary fiber intake (g/day)	12.4 ± 4.2	12.8 ± 4.5	0.582
Glucose metabolism			
Fasting glucose (mmol/L)	5.24 ± 0.68	5.28 ± 0.72	0.742
Fasting insulin (μU/mL)	12.4 ± 4.8	12.8 ± 5.2	0.628
HOMA-IR	2.89 ± 1.12	3.02 ± 1.18	0.512
Lipid profile			
Total cholesterol (mmol/L)	5.12 ± 0.86	5.18 ± 0.92	0.684
LDL-C (mmol/L)	3.24 ± 0.72	3.28 ± 0.78	0.748
HDL-C (mmol/L)	1.32 ± 0.28	1.28 ± 0.26	0.382
Triglycerides (mmol/L)	1.48 ± 0.62	1.52 ± 0.68	0.714
Inflammatory markers			
hs-CRP (mg/L)	2.14 ± 1.23	2.08 ± 1.18	0.768
IL-6 (pg/mL)	3.42 ± 1.56	3.38 ± 1.49	0.876
TNF-α (pg/mL)	8.76 ± 2.84	8.62 ± 2.71	0.762
IL-10 (pg/mL)	4.28 ± 1.64	4.35 ± 1.72	0.803
Gut microbiota diversity			
Shannon index	3.82 ± 0.48	3.79 ± 0.51	0.716
Chao1 index	286.4 ± 42.8	289.2 ± 45.3	0.702
Simpson index	0.91 ± 0.04	0.90 ± 0.05	0.684

Data are presented as mean ± standard deviation or *n* (%). hs-CRP, high-sensitivity C-reactive protein; IL-6, interleukin-6; TNF-α, tumor necrosis factor-alpha; IL-10, interleukin-10; HOMA-IR, Homeostatic Model Assessment for Insulin Resistance, calculated as fasting glucose (mmol/L) × fasting insulin (μU/mL)/22.5; LDL-C, low-density lipoprotein cholesterol; HDL-C, high-density lipoprotein cholesterol.

### 3.2 Primary outcome: changes in gut microbiota diversity

All subsequent analysis was performed using data from the subjects who participated in the 24-week assessment (intervention, *n* = 58; control, *n* = 61), unless otherwise stated. Results from the analysis of composition of gut microbiota show there was significant improvement in the treatment group compared with the control subjects during the 24-week period. Alpha diversity metrics indicated there was progressive increase in the diversity among subjects treated with the combined functional dietary fiber and at-home exercise treatment, depicted in Table 2. Shannon diversity indices, as identified as the main measure of treatment outcomes, significantly increased in the treatment group from 3.82 ± 0.48 at baseline to 4.08 ± 0.52 at week 12, and then to 4.31 ± 0.49 at week 24, corresponding to an overall increase of 0.49 units. In comparison, there was little change in the treatment-naïve controls whose Shannon indices remained relatively constant throughout the duration of the trial period. Linear analysis using the linear model equation from the linear mixed model analysis demonstrated there was significant time-by-group interaction affecting the Shannon indices (*p* < 0.001), suggesting important changes in the combined functional dietary fiber and at-home exercise treatment beyond natural changes.

**Table 2:** Changes in gut microbiota alpha diversity indices.

Variable	Group	Baseline	Week 12	Week 24	Change (0–24w)	*p* (time × group)
Shannon index	Intervention	3.82 ± 0.48	4.08 ± 0.52	4.31 ± 0.49	+0.49 ± 0.21	<0.001
	Control	3.79 ± 0.51	3.82 ± 0.49	3.85 ± 0.53	+0.06 ± 0.18	
Chao1 index	Intervention	286.4 ± 42.8	308.6 ± 45.2	329.2 ± 48.6	+42.8 ± 22.4	<0.001
	Control	289.2 ± 45.3	292.8 ± 44.1	297.6 ± 46.8	+8.4 ± 19.6	
Simpson index	Intervention	0.91 ± 0.04	0.93 ± 0.03	0.95 ± 0.03	+0.04 ± 0.02	<0.001
	Control	0.90 ± 0.05	0.91 ± 0.04	0.91 ± 0.05	+0.01 ± 0.02	
Observed species	Intervention	312.5 ± 48.6	338.2 ± 52.4	362.8 ± 54.2	+50.3 ± 24.8	<0.001
	Control	315.8 ± 50.2	319.4 ± 49.8	324.2 ± 51.6	+8.4 ± 20.2	

Data are presented as mean ± standard deviation. *p*-values represent the significance of time-by-group interaction effects from linear mixed-effects models.

Similar trends were seen in other alpha diversity indices. The Chao1 richness measure, which represents the estimated number of bacterial species, was increased by 42.8 units in the treatment group compared to a modest increase of 8.4 units in the control group. The Simpson diversity index, which takes both richness and diversity into account, was also found to have significant improvements in the treatment group. These data together imply that the combined treatment had positively impacted both richness and diversity in the gut microbial composition.

**Figure 2:**
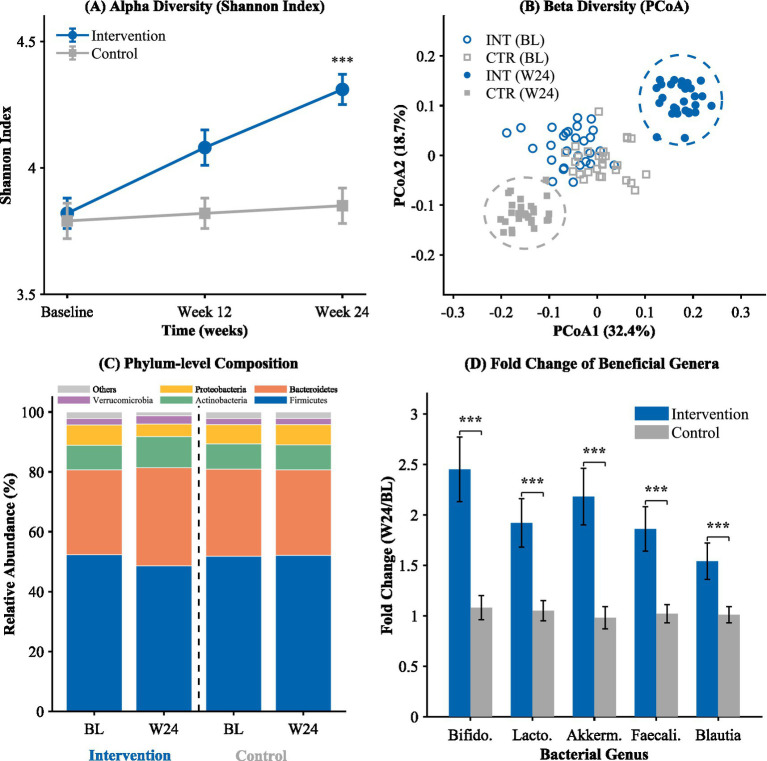
Effects of the combined intervention on gut microbiota diversity and taxonomic composition. **(A)** Alpha diversity (Shannon Index); **(B)** Beta diversity (PCoA); **(C)** Phylum-level composition; **(D)** Fold change of beneficial genera.

Beta diversity analysis performed through principal coordinate analysis using Bray–Curtis dissimilarity indicated the existence of different patterns of clusters between the groups at the end of the study, as shown in Figure 2. At baseline, there was significant overlap in both groups’ samples, suggesting the existence of similar microbial profiles. At week 24, the samples in the intervention group demonstrated large changes in the first coordinate axis, showing significant separation from the other group. Permutational multivariate analysis of variance in the composition data at week 24 further supported significant differences in the total composition of microbiota between the two groups (*p* = 0.001). Taxonomic analysis revealed significant changes in the intervention group: beneficial bacteria increased substantially—Bifidobacterium (+104.8%, from 4.2 to 8.6%), *Faecalibacterium prausnitzii* (+62.1%), Akkermansia (+128.6%), and Lactobacillus (+100.0%) (all *p* < 0.001). Conversely, potentially pathogenic bacteria decreased—Escherichia-Shigella (−50.0%) and Enterobacteriaceae (−46.2%) (all *p* < 0.001).

### 3.3 Primary outcome: changes in inflammatory markers

Systemic inflammation markers showed significant improvements in the treatment group compared to the control group during the 24-week duration of the trial, as shown in Table 3. High-sensitivity C-reactive protein, the main inflammation marker, was significantly lowered in the treatment group consisting of combined functional dietary fiber and home exercise, reducing from 2.14 ± 1.23 mg/L at baseline to 1.24 ± 0.82 mg/L at week 24, showing a reduction of 42.1% in comparison to baseline. Based on cardiovascular risk categories (low: < 1.0 mg/L; intermediate: 1.0–3.0 mg/L; high: > 3.0 mg/L) (7), the proportion of intervention participants in the low-risk category increased from 28.6 to 62.1%, compared with minimal change in controls (29.5 to 32.8%). However, hs-CRP in the control group remained constant with less variation in the duration of the trial. Linear mixed models analysis demonstrated significant interaction between hs-CRP and time factors in the treatment and control groups (*p* < 0.001), validating that the treatment was able to reduce hs-CRP significantly, an important inflammation marker.

**Table 3:** Inflammatory marker concentrations at each timepoint.

Marker	Timepoint	Intervention (*n* = 58)	Control (*n* = 61)	Between-group *p*
hs-CRP (mg/L)	Baseline	2.14 ± 1.23	2.08 ± 1.18	0.768
	Week 12	1.68 ± 0.98	2.04 ± 1.15	0.042
	Week 24	1.24 ± 0.82	2.02 ± 1.12	<0.001
IL-6 (pg/mL)	Baseline	3.42 ± 1.56	3.38 ± 1.49	0.876
	Week 12	2.78 ± 1.32	3.34 ± 1.45	0.018
	Week 24	2.21 ± 1.08	3.30 ± 1.42	<0.001
TNF-α (pg/mL)	Baseline	8.76 ± 2.84	8.62 ± 2.71	0.762
	Week 12	7.42 ± 2.48	8.68 ± 2.75	0.006
	Week 24	6.26 ± 2.12	8.76 ± 2.78	<0.001
IL-10 (pg/mL)	Baseline	4.28 ± 1.64	4.35 ± 1.72	0.803
	Week 12	4.92 ± 1.78	4.38 ± 1.70	0.065
	Week 24	5.64 ± 1.92	4.42 ± 1.68	<0.001

Data are presented as mean ± standard deviation. Between-group *p*-values were calculated using independent *t*-tests. hs-CRP, high-sensitivity C-reactive protein; IL-6, interleukin-6; TNF-α, tumor necrosis factor-alpha; IL-10, interleukin-10.

Patterns of improvement were found consistently in other pro-inflammatory cytokines. The reduction in interleukin-6 level was found to be 35.4% in the intervention group, which was significantly different from 2.4% in the control group (*p* < 0.001). Similar was the case with tumor necrosis factor-alpha, where there was a reduction of 28.6% in the intervention group, whereas there was an increase of 1.6% in the control group (*p* < 0.001). The reduction in these pro-inflammatory biomarkers was associated with an increase in the level of the anti-inflammatory cytokine interleukin-10, whose level increased by 31.8% in the intervention group, whereas in the control group, there was no significant change (*p* < 0.001).

**Figure 3:**
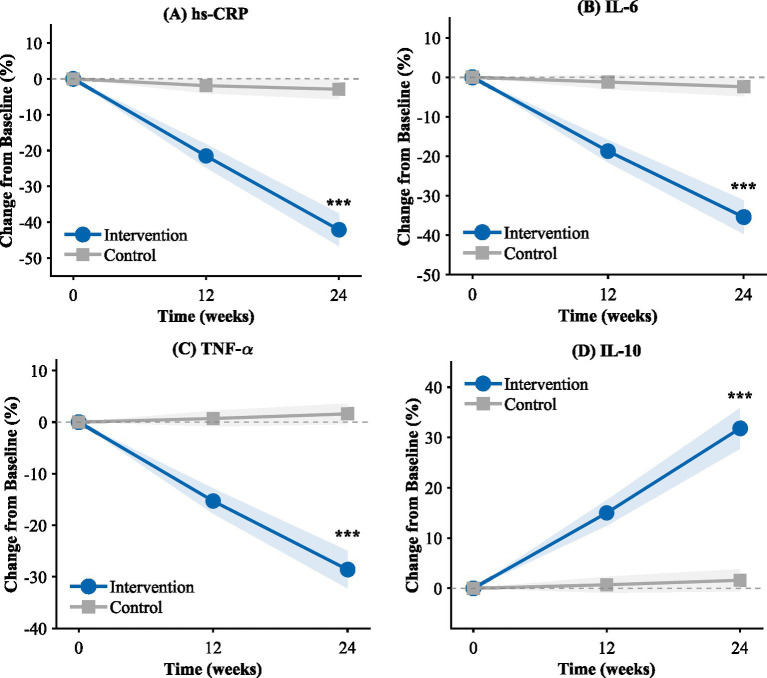
Percentage change in inflammatory markers from baseline. **(A)** hs-CRP; **(B)** IL-6; **(C)** TNF-α; **(D)** IL-10.

The changes in all inflammation factors from baseline are shown in Figure 3. For all four cytokines, the changes developed incrementally over time, becoming greater from week 12 through week 24. The intergroup differences in all factors proved to be significant at both times. In conclusion, these data show that the combined treatment was effective in reducing low-grade inflammation in sedentary urban citizens, and these changes were measurable through several inflammation pathways.

### 3.4 Secondary outcomes

The combined treatment resulted in positive changes in several secondary endpoints, as shown in Table 4. Parameters of body composition indicated significant changes in the treatment group during the 24-week period. Body fat percentage was reduced from 28.4 ± 5.6% at baseline to 25.8 ± 5.2% at week 24 in the treatment group, with a reduction of 2.6% points; in the control group, there was little change. At the same time, there was an increase in lean body mass in the treatment group due to the anabolic effects of the home-based resistance exercise component. Waist circumference, as an important measure of abdominal obesity, was reduced by 4.2 cm in the treatment group compared to 0.4 cm in the control group; there was a highly significant interaction between time and treatment (*p* < 0.001).

**Table 4:** Changes in secondary outcome measures.

Variable	Group	Baseline	Week 12	Week 24	Change (0–24w)	*p* (time × group)
Body composition						
Body fat (%)	Intervention	28.4 ± 5.6	27.0 ± 5.4	25.8 ± 5.2	−2.6 ± 1.8	<0.001
	Control	28.8 ± 5.3	28.6 ± 5.4	28.5 ± 5.4	−0.3 ± 1.2	
Lean mass (kg)	Intervention	48.2 ± 8.6	48.8 ± 8.7	49.4 ± 8.8	+1.2 ± 1.4	0.008
	Control	47.8 ± 8.4	47.8 ± 8.4	47.9 ± 8.5	+0.1 ± 1.0	
Waist circumference (cm)	Intervention	86.2 ± 9.4	84.2 ± 9.0	82.0 ± 8.8	−4.2 ± 2.6	<0.001
	Control	86.6 ± 9.1	86.4 ± 9.0	86.2 ± 9.0	−0.4 ± 1.8	
BMI (kg/m^2^)	Intervention	24.7 ± 2.8	24.2 ± 2.7	23.8 ± 2.6	−0.9 ± 0.8	<0.001
	Control	24.9 ± 2.6	24.9 ± 2.6	24.8 ± 2.7	−0.1 ± 0.6	
Physical activity						
Sitting time (h/day)	Intervention	9.1 ± 1.4	8.2 ± 1.3	7.8 ± 1.3	−1.3 ± 0.8	<0.001
	Control	9.3 ± 1.5	9.2 ± 1.5	9.1 ± 1.4	−0.2 ± 0.6	
MVPA (min/week)	Intervention	62.4 ± 38.5	142.6 ± 42.8	168.2 ± 45.6	+105.8 ± 42.3	<0.001
	Control	58.6 ± 35.2	60.4 ± 36.8	64.2 ± 38.4	+5.6 ± 28.4	
Subjective measures						
MFI-20 total score	Intervention	58.4 ± 12.6	50.8 ± 11.4	45.2 ± 10.8	−13.2 ± 8.4	<0.001
	Control	57.8 ± 11.8	57.2 ± 12.0	56.4 ± 12.2	−1.4 ± 6.2	
SF-36 PCS	Intervention	48.2 ± 8.4	51.8 ± 8.0	54.6 ± 7.8	+6.4 ± 5.2	<0.001
	Control	47.6 ± 8.2	47.8 ± 8.1	48.2 ± 8.0	+0.6 ± 4.8	
SF-36 MCS	Intervention	46.8 ± 9.2	50.2 ± 8.8	52.4 ± 8.6	+5.6 ± 4.8	<0.001
	Control	47.2 ± 8.8	47.4 ± 8.9	47.8 ± 9.0	+0.6 ± 5.2	
GSRS total score	Intervention	8.4 ± 4.2	8.0 ± 4.0	7.8 ± 3.8	−0.6 ± 2.4	0.342
	Control	8.2 ± 4.0	8.1 ± 4.0	8.0 ± 4.1	−0.2 ± 2.2	

Data are presented as mean ± standard deviation. BMI, body mass index; MVPA, moderate-to-vigorous physical activity; MFI-20, Multidimensional Fatigue Inventory (lower scores indicate less fatigue); SF-36 PCS/MCS, Short Form-36 Physical/Mental Component Summary (higher scores indicate better quality of life); GSRS, Gastrointestinal Symptom Rating Scale (lower scores indicate fewer symptoms).

Patterns of sedentary behavior demonstrated significant changes after the intervention. The reduction in sedentary behavior was quantified using accelerometry and valid sedentary behavior measures, and there was a significant reduction in sitting behavior from 9.1 ± 1.4 h per day at baseline to 7.8 ± 1.3 h at week 24 in the intervention group, which translates to about 78 min per day. The reduction in sedentary behavior was further complemented by increased moderate to vigorous physical activity, which was shown to increase from 62.4 ± 38.5 min per week to 168.2 ± 45.6 min per week in the intervention group.

The scores from the subjective well-being measurements also showed significant changes. In the Multidimensional Fatigue Inventory, total scores were positively affected in the intervention group, where there was a large reduction from 58.4 ± 12.6 to 45.2 ± 10.8. These changes reflect reduced fatigue in terms of physical, mental, and motivational aspects. Health-related quality-of-life scores, which are obtained from the SF-36 Health Survey, showed significant increases in both the Physical Component Summary scores (48.2 ± 8.4 to 54.6 ± 7.8) and the Mental Component Summary scores (46.8 ± 9.2 to 52.4 ± 8.6) in the intervention group. GI symptom scores remained constant in both the intervention and the control groups all through the trial period, supporting the observation that the supplement protocol was tolerated without any adverse reactions concerning digestive comfort. These results make it clear that the combined treatment protocol resulted in comprehensive benefits to the recipients’ health, spreading beyond the established outcome variables of increased diversity of the microbial population in the guts and reduction of inflammation. The intervention also improved metabolic parameters. Fasting glucose decreased by 6.1% in the intervention group versus 0.8% in controls (P time × group = 0.008). HOMA-IR decreased by 22.5% versus 2.6% (*p* < 0.001). Total cholesterol decreased by 8.2% versus 1.4% (*p* = 0.004), and triglycerides decreased by 15.5% versus 3.2% (*p* = 0.001).

### 3.5 Mechanistic exploration: microbiota-inflammation association analysis

To explain the possible mechanisms associated with the positive changes in both diversity and inflammation, the current trial explored comprehensive correlation and mediation analysis. The level of short-chain fatty acids in feces had shown remarkable increases in the treatment group, as shown in Table 5. The level of acetate, propionate, and butyrate was found to increase significantly in the treatment group after the 24-week period, with greater increases in butyrate from 12.4 ± 4.8 μmol/g at baseline to 18.6 ± 5.2 μmol/g at week 24, indicating a 50% increase. In comparison, there was merely a 4.8% increase in the treatment group than in the other group in terms of the overall increase in the level of total short-chain fatty acids at 38.2% in the treatment group. There was significant reduction in intestinal barrier function as evidenced by the decline in serum zonulin in addition to the reduction in the level of lipopolysaccharide-binding protein in the treatment group.

**Table 5:** Changes in short-chain fatty acids and gut barrier markers.

Variable	Group	Baseline	Week 24	Change (%)	*p* (time × group)
Fecal SCFAs (μmol/g)					
Acetate	Intervention	48.2 ± 12.6	62.4 ± 14.8	+29.5%	<0.001
	Control	47.8 ± 11.8	49.2 ± 12.4	+2.9%	
Propionate	Intervention	14.6 ± 4.2	19.8 ± 5.4	+35.6%	<0.001
	Control	14.2 ± 4.0	14.8 ± 4.2	+4.2%	
Butyrate	Intervention	12.4 ± 4.8	18.6 ± 5.2	+50.0%	<0.001
	Control	12.2 ± 4.5	12.8 ± 4.6	+4.9%	
Total SCFAs	Intervention	75.2 ± 18.4	103.9 ± 22.6	+38.2%	<0.001
	Control	74.2 ± 17.8	77.8 ± 18.2	+4.8%	
Gut barrier markers					
Zonulin (ng/mL)	Intervention	48.6 ± 14.2	38.4 ± 12.8	−21.0%	<0.001
	Control	47.8 ± 13.8	46.2 ± 14.0	−3.3%	
LBP (μg/mL)	Intervention	12.8 ± 3.6	9.6 ± 3.2	−25.0%	<0.001
	Control	12.4 ± 3.4	12.0 ± 3.5	−3.2%	

Data are presented as mean ± standard deviation. SCFAs, short-chain fatty acids; LBP, lipopolysaccharide-binding protein. *p*-values represent the significance of time-by-group interaction effects from linear mixed-effects models.

**Figure 4:**
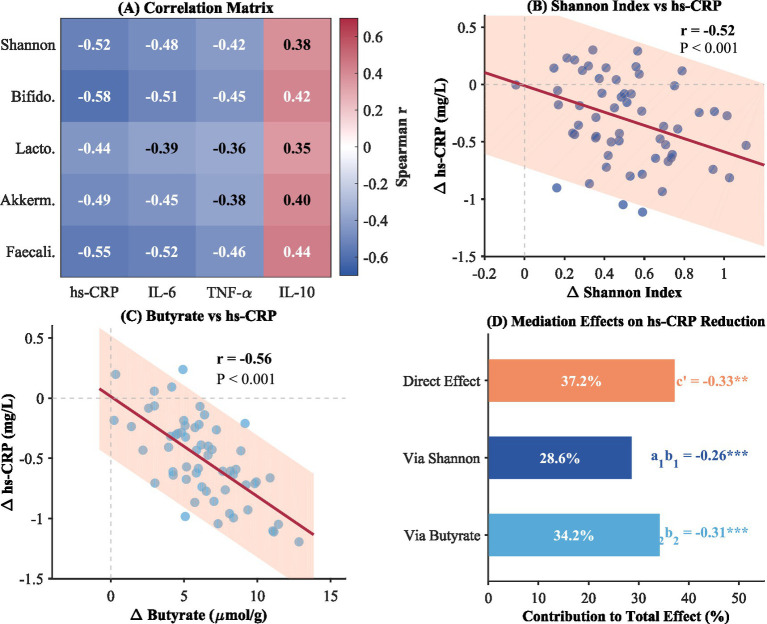
Microbiota-inflammation association and mediation analysis. **(A)** Correlation matrix; **(B)** Shannon index vs. hs-CRP; **(C)** Butyrate (μmol/g); **(D)** Contribution to total effect (%).

Correlation analysis indicated significant relationships between changes in microbiota parameters and decreases in inflammation markers, as shown in Figure 4. The Shannon diversity index change displayed strong negative correlations with changes in high-sensitivity C-reactive protein (*r* = −0.52, *p* < 0.001), suggesting that greater increases in diversity were related to larger decreases in systemic inflammation. However, this correlation cannot establish causality; the observed relationship may reflect direct effects, reverse causation, or shared upstream factors. Similar patterns of inverse correlations appeared when changes in the Shannon diversity index were related to changes in interleukin-6 and tumor necrosis factor-alpha. Increases in bacterial genus Bifidobacterium and *Faecalibacterium prausnitzii* displayed strong negative correlations with changes in inflammation markers.

Further support for the mediator role of SCFAs was obtained from the mediation analysis. Butyrate was found to significantly mediate the relationship between the intervention and the reduction in hs-CRP, explaining about 34.2% of the total effect. The indirect pathway of Shannon diversity index was found to explain an additional 28.6% of the total effect of the intervention on the reduction in inflammation markers. These pieces of evidence, taken together, implicate the hypothesis that the combined dietary fiber and exercise intervention works, at least in part, through the adjustment of the composition of the gut microbiota and increased SCFAs.

### 3.6 Subgroup analyses

**Figure 5:**
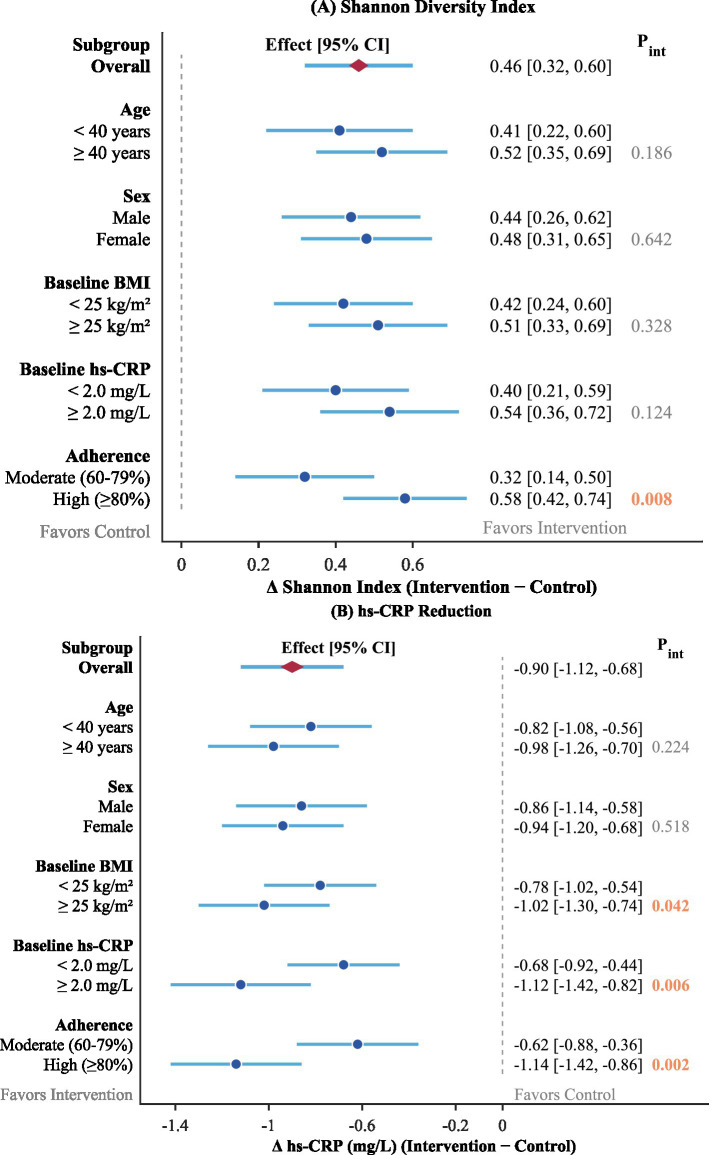
Subgroup analyses of intervention effects on primary outcomes. **(A)** Shannon diversity index; **(B)** hs-CRP reduction.

Pre-specified subgroup analyses were further performed to explore whether there was heterogeneity in the treatment effects of the primary outcomes according to the characteristics of the participants, as shown in Figure 5. Each subgroup shown in the forest plot in Figure 5 presents the treatment effect of Shannon diversity index change and reduction in hs-CRP levels based on the subgroup characteristics.

The benefits of the intervention remained consistent in all subgroups, and all point estimates indicated benefits in the intervention group. Subgroup analysis based on age demonstrated that, in patients who were 40 years or older, there was a numerically greater reduction in Shannon diversity index (0.52 vs. 0.41) and hs-CRP (−0.98 vs. −0.82) in the intervention compared with the usual care group; however, these interactions failed to reach significance (*p* = 0.186 and *p* = 0.224, respectively). Subgroup analysis based on gender was found to have non-significant effect modifications in both endpoints.

Baseline metabolic condition seemed to affect the extent of responses to interventions. Patients with increased baseline BMI (≥25 kg/m^2^) had greater hs-CRP reduction than those with normal BMI (−1.02 vs. -0.78 mg/L, Pinteraction = 0.042), indicating that patients with increased inflammation due to obesity could potentially gain increased benefits from the combined treatment. Correspondingly, patients with increased baseline hs-CRP levels (≥2.0 mg/L) had greater inflammatory marker reduction than those with lower baseline hs-CRP levels, suggesting an apparent regression towards the mean with greater potential for reduction in patients with increased inflammation.

Adherence to the intervention was found to be an important modifier of both outcomes. In fact, subjects who presented high adherence (>80% compliance in both dietary fiber ingestion and exercise performance) experienced much larger decreases in Shannon diversity index and hs-CRP than those who reported moderate adherence (60–79%), with significant interaction terms for both in the model (Pinteraction < 0.01). These data highlight the role of long-term active participation in achieving maximal benefits from interventions and that there might exist relations between compliance and outcomes.

## 4 Discussion

In the current RCT, it was found that the combined treatment approach involving functional dietary fiber supplements and exercise at home resulted in significant changes in the diversity of gut microbiota and reduction in systemic inflammation in sedentary adults. The current evidence adds to the existing body of knowledge by demonstrating the combined effects between dietary and exercise components in modulating the link between gut and inflammation in subjects who exhibit high sitting times due to increased cardiometabolic risk. While the study design does not allow definitive attribution of effects to individual intervention components, comparison with previous single-intervention studies provides preliminary insights. Meta-analyses of dietary fiber interventions alone typically report Shannon diversity increases of 0.15–0.25 units over 12–24 weeks (18), while exercise-only interventions in sedentary adults have shown more modest diversity changes of 0.08–0.18 units (26, 27). The 0.49-unit increase observed in the current combined intervention exceeds these individual estimates, suggesting potentially additive or greater-than-additive effects. Similarly, hs-CRP reductions from fiber-only interventions typically range from 15 to 25% (18), while exercise-only interventions report 10–20% reductions (24). The 42.1% reduction observed in our study substantially exceeds these individual estimates.

The finding of increased alpha diversity in the gastrointestinal microbiome, as estimated using the Shannon index, corresponds to other studies investigating the independent role of dietary fiber and exercise in modulating microbiomes. In a prospective analysis of sedentary subjects, exercise alone was shown to result in small but potentially beneficial changes in gastrointestinal microbiomes, albeit much less than in the current analysis (26). In related studies focusing on exercise-induced modifications in gastrointestinal microbiomes, it was found that both lean and obese volunteers responded in different ways towards exercise regimens, where changes in microbial diversity had corresponding alterations in body composition (27). The current study confirms these other findings in showing that the addition of both exercise regimens and prebiotic fiber supplements produce combined effects resulting in larger changes in microbial diversity rather than in other analytical estimates relevant to either exercise or supplements.

These combined effects are most likely due to complementary mechanisms in the gut microbiota. Dietary fibers provide fermentation substrates for beneficial bacteria and facilitate gut homeostasis through metabolite-sensing receptors such as GPR43 and GPR109A (16, 28). The strong rises in the level of SCFAs documented in the intervened group support these postulates, since SCFAs are the key intermediates in fiber-related health benefits. Butyrate, identified to have the most intense increase in this analysis, has anti-inflammatory activities through the activation of G protein–coupled receptors GPR43 and GPR109A, leading to the inhibition of NF-κB and the suppression of pro-inflammatory cytokines (29). Rather than affecting the microbiota composition or fermentation activity directly, the likely mechanisms underlying the reduction in the systemic inflammation level in accordance with fiber and exercise-induced changes in fermentation activity could therefore involve these receptor mechanisms.

The anti-inflammatory responses observed in this trial are in line with emerging evidence about the role of lifestyle interventions in the modulation of chronic low-grade inflammation. Exercise-induced suppression of inflammation has been shown to occur through several mechanisms, including the secretion of anti-inflammatory myokines from contracting skeletal muscle and changes in adipose tissue metabolism (21, 23). These exercise-induced responses appear to be potentiated when complemented with dietary modifications that promote optimal gut microbial activity. Although systematic reviews assessing the modification of gut microbiota through physical activity have reported inconsistent findings, these have been likely due to differences in exercise protocols and population characteristics (22, 30). It would appear that the organized nature of the exercise intervention implemented in the current study, in addition to the prebiotic activity provided through functional fiber supplements, helped to mediate the enhanced anti-inflammatory activity observed.

The changes in high-sensitivity C-reactive protein and other inflammatory cytokines in our trial have significant clinical implications in the prevention of cardiometabolic disease. In fact, the most recent meta-analysis of exercise training programs in sedentary individuals identified improvements in glucose metabolism and inflammation, although with generally small interventional effect sizes (24). The extent of reduction in the level of inflammation obtained in our trial far surpasses the extent observed in many previous inflammation-targeting lifestyle interventions, although it likely represents the combined benefits of concurrently targeting dietary and physical activity behavior domains. Indeed, there appears to be important evidence suggesting that multicomponent interventions can target inflammation to greater meaningful extent than single-interventional components.

In terms of practical application of the intervention protocol, there are aspects related to public health translation that warrant discussion. Home-based exercise programs have already demonstrated efficacy in providing congruent benefits to center-based exercise programs, coupled with benefits in accessibility and long-term compliance (31, 32). The fact that there was an equally high level of retention and good level of compliance in this trial suggests that the total treatment program has the potential to benefit sedentary individuals who could potentially face challenges in accessing conventional gym exercise programs. The fiber supplement part of the treatment program, which included resistant starch, inulin, and beta-glucan combined, was aimed at maximizing prebiotic benefits without any adverse gastrointestinal symptoms due to dose-escalation considerations. Previous studies have already indicated significant individual variations in responses to prebiotic supplements based on individual dietary fiber intake and baseline composition of the gastrointestinal microbiota (33), potentially explaining the disparities in individual trial outcomes.

The correlation and mediation test offer preliminary data supporting the mechanistic role of the gut microbiata in mediating the anti-inflammatory responses of the treatment. The observation that changes in Shannon diversity index negatively correlated with changes in inflammation indices suggests that the normalization of microbial composition contributes to anti-inflammatory responses. Butyrate accounts for approximately one-third of the changes in high-sensitivity c-reactive protein in mediating the treatment, and its anti-inflammatory role has long been supported by scientific evidence (10). The current observation fits well into the paradigm suggesting that the microbiata can be considered as a target in preventing inflammation-related chronic diseases. However, the current observation lacks the capability to imply causations due to the nature of cross-sectional analysis in the current observation.

Subgroup analysis has shown that these intervention effects are moderated by baseline metabolic status and by levels of adherence. In both cases, greater benefits of metabolic changes have been found in those who had high baseline BMI and inflammation, in line with the hypothesis that patients with less favorable characteristics at baseline have greater potential benefits (14, 15). The fact that the level of adherence turned out to be significant as an effect modifier in these perioperative metabolic interventions underlines the significance of active behavioral participation in achieving optimal outcomes in terms of personal health. This study makes several novel contributions to the existing literature. First, it specifically targets sedentary urban adults—a population characterized by both high cardiometabolic risk and unique microbiota profiles shaped by modern urban lifestyles (1, 2). Second, the specific multi-fiber formulation combining RS2, inulin, and beta-glucan represents a mechanistically informed approach designed to target complementary bacterial populations, unlike single-fiber studies (14, 33). Third, the home-based exercise protocol with high retention (82.9%) and adherence (82.8%) demonstrates feasibility for real-world implementation in populations who may face barriers to gym-based programs (31, 32). Fourth, the comprehensive mechanistic exploration—integrating SCFA quantification, gut barrier markers, and mediation analysis—provides deeper understanding of the intervention’s biological pathways than typically available in lifestyle intervention trials.

Several limitations of the study need to be acknowledged. First, the lack of a factorial design prevents attribution of effects to individual intervention components; we cannot determine whether the observed effects are truly synergistic, simply additive, or primarily driven by one component. Future studies with 2 × 2 factorial designs including fiber-only and exercise-only arms are essential to disentangle these contributions (34–36). Second, the open-label nature of the trial introduces potential performance and detection biases, though the use of objective biological outcomes (microbiota sequencing, blood biomarkers, fecal SCFAs) partially mitigates these concerns. Third, adherence monitoring relied partially on self-reported measures despite our dual verification approach with wearable devices. Fourth, the homogeneous population from a single urban setting in South Korea limits generalizability to other populations with different ethnic backgrounds, dietary patterns, or baseline microbiota compositions (37). Fifth, the 24-week duration does not address long-term sustainability of benefits following intervention cessation. Sixth, while mediation analysis supports the role of SCFAs in mediating anti-inflammatory effects, this statistical approach cannot establish true causality.

It would be important to cover these issues in future studies using several strategies. Using factorial trial designs, it would be possible to distinguish between the independent and combined (interaction) effects of fiber and exercise in order to detect any interaction effects (36). Longer follow-up times after the active treatment period would help to unravel whether the positive changes found in the composition of the gut microbiota and inflammation are sustained in the long run. The analysis of individual differences in treatment responses, using baseline microbiome analysis to define predictors of treatment success, would offer an interesting future perspective regarding tailored treatment strategies (37). It would offer important new evidence to support exercise-mediterranean diet interventions targeting the gut-inflammation link in other at-risk population subsets, such as the elderly or subjects with metabolic syndrome.

## 5 Conclusion

Addressing our primary research questions, this 24-week randomized controlled trial demonstrates that the combined functional dietary fiber supplementation and home-based exercise intervention significantly increases gut microbiota diversity (Shannon index: +0.49 units, +12.8%, *p* < 0.001) and reduces systemic inflammation (hs-CRP: −42.1%, *p* < 0.001) in sedentary urban adults compared to usual lifestyle controls. The current randomized controlled trial offers evidence to support the effectiveness of the 24-week combined functional dietary fiber supplement and home-based exercise program in reducing systemic low-grade inflammation and restoring diversity to the dysfunctional gastrointestinal microbiome in sedentary men and women in an urban setting. Indeed, there was a significant increase in the Shannon diversity index from 3.82 to 4.31 post-intervention in the treatment arm, in addition to significant reductions in markers of inflammation such as high-sensitivity CRP (42.1% reduction), interleukin-6 (35.4% reduction), and tumor necrosis factor alpha (28.6% reduction). These positive changes further correlated with increased fecal butyrate (50% increase) and significant decreases in indices of permeability. These data suggest that combined dietary and exercise modifications aimed at the gastrointestinal microbiome offer an important and practical means of reducing cardiometabolic risk in sedentary men and women.

## Data Availability

The raw 16S rRNA gene sequencing data have been deposited in the NCBI Sequence Read Archive (SRA) under accession number PRJNA1429892. All processed datasets, analysis scripts, SCFA measurements, inflammatory biomarker data, anthropometric datasets, and questionnaire results are included in the Zenodo repository: https://doi.org/10.5281/zenodo.18798564 (DOI: 10.5281/zenodo.18798564).
